# A Pacific needs analysis model: a proposed methodology for assessing the needs of facility-based emergency care in the Pacific region

**DOI:** 10.1186/s12913-020-05398-w

**Published:** 2020-06-19

**Authors:** Georgina Phillips, Kathryn Bowman, Trina Sale, Gerard O’Reilly

**Affiliations:** 1 School of Public Health and Preventive Medicine, Monash University, 553 St. Kilda Rd., Melbourne, VIC 3004 Australia; 2 Emergency Department, St Vincent’s Hospital Melbourne, Melbourne, Australia; 3 Hospital Independence Program, St Vincent’s Hospital Melbourne, Melbourne, Australia; 4 Emergency Department, National Referral Hospital, Honiara, Solomon Islands; 5 Emergency and Trauma Centre, The Alfred Hospital, Melbourne, Australia

**Keywords:** Emergency medical services, Pacific Islands, Needs assessments, Health services research

## Abstract

**Background:**

Emergency care (EC) describes team-based, multidisciplinary clinical service provision, advocacy and health systems strengthening to address all urgent aspects of illness and injury for all people. In order to improve facility-based EC delivery, a structured framework is necessary to outline current capacity and future needs. This paper draws on examples of EC Needs Assessments performed at the national hospitals of three different Pacific Island Countries (PICs), to describe the development, implementation and validation of a structured assessment tool and methodological approach to conducting an EC Needs Assessment in the Pacific region.

**Methods:**

This is a retrospective, descriptive analysis of the development of the Pacific Emergency Care Assessment (PECA) table using patient-focused principles within an EC systems framework. Tool implementation occurred through observation, literature review and interviews using a strengths-based, action-research and ethnographic methodological approach in Timor-Leste, Kiribati and the Solomon Islands. The 2014 Solomon Islands EC Needs Assessment provides the main context to illustrate and discuss the overall conduct, feasibility, validity and reliability of the PECA tool and methodological approach.

**Results:**

In each site, the methodological implementation enabled completion of both the PECA table and comprehensive report within approximately 6 weeks of first arriving in country. Reports synthesising findings, recommendations, priority action areas and strategies were distributed widely amongst stakeholders. Examples illustrate Face and Content, Construct and Catalytic validity, including subsequent process and infrastructure improvements triggered by the EC Needs Assessment in each site. Triangulation of information and consistency of use over time enhanced reliability of the PECA tool.

Compared to other EC assessment models, the Pacific approach enabled rich data on capacity and real-life function of EC facilities. The qualitative, strengths-based method engenders long-term partnerships and positive action, but takes time and requires tailoring to a specific site.

**Conclusion:**

In PICs and other global contexts where EC resources are underdeveloped, a PECA-style approach to conducting an EC Needs Assessment can trigger positive change through high local stakeholder engagement. Testing this qualitative implementation method with a standardised EC assessment tool in other limited resource contexts is the next step to further improve global EC.

## Background

All people may experience unexpected illness or injury that requires urgent health care interventions to prevent death or disability. The term ‘emergency care’ (EC) encompasses such interventions and can be defined as ‘multidisciplinary, team-based prevention and clinical service provision, capacity development and health systems strengthening to handle acute and urgent aspects of all illness and all injuries’ [1]. Effective EC can occur in hospitals, clinics and in the community ‘pre-hospital’ setting. In facilities, the safe and effective provision of EC requires a simple organised system that includes trained staff, core processes (such as triage), an appropriate environment and basic equipment [2].

Globally, EC is poorly understood and EC systems frequently absent [3]. Yet evidence exists that rapid interventions, even in low resource environments, can improve patient outcomes and address several of the health-related Sustainable Development Goals [4–7].

In order to understand what may be required at a healthcare facility to improve EC, a structured framework for measuring EC capacity and future needs is necessary. There are various published models of how to construct a healthcare Needs Assessment [8–12], but very few specifically address EC and EC systems. Aside from highly refined methods and tools used by global agencies in disaster, mass casualty and complex humanitarian crisis situations [13, 14], assessing routine needs for daily EC in low resource environments is not well defined. Adapting from the Wright et al. [10] definition of a Health Needs Assessment, we define an EC Needs Assessment as.

*‘the systematic approach to ensuring a health service uses resources efficiently and effectively to improve the health outcomes of all patients with acute and urgent illness and injury. It employs quantitative and qualitative methods to describe emergency care status and current emergency care problems, identify gaps in emergency care delivery, and determine priorities for emergency care improvement according to the local resource environment.’*

Current work from Africa [15] and by the World Health Organisation (WHO) [16] provide examples of assessment tools that have been applied during structured, focused EC Needs Assessments. Some examples in the literature describe how tools are utilised in a wider methodological approach [17] to reach conclusions about EC gaps and priorities for future EC capacity development work. In the Pacific region, national governments desire improvements in EC delivery, but lack knowledge of how to prioritise and proceed with developing EC capacity and function. At the request of local Ministries of Health, EC Needs Assessments accompanied by recommendations have been completed at the national hospital in three Pacific Island Countries (PICs); Timor-Leste (2009), Kiribati (2011) and the Solomon Islands (2014).

The aim of this paper is to describe the development, implementation and initial validation of a structured EC assessment tool within the wider context of an action-research and ethnographic methodological approach to conducting an EC Needs Assessment in the Pacific region.

## Methods

This is a retrospective, descriptive analysis of the development and implementation of the Pacific Emergency Care Assessment (PECA) tool in three settings in the Pacific region, with an emphasis on the most recent setting, the Solomon Islands, in 2014. The method adopted in each country combined an action-research framework with semi-structured interviews and the structured PECA tool to clarify local priorities and appropriate strategies for EC improvement. Outcomes of PECA tool application using the Solomon Islands example are illustrated and discussed in order to demonstrate feasibility, validity and reliability.

### Setting

The Pacific is a unique region, characterised by small populations dispersed across islands living in high urban density and remote rural villages, with limited human resources and medium to low human development (according to the United Nations Human Development Index) [18].

PICs bear the double burden of non-communicable [19] and communicable diseases, as well as a high rate of trauma and interpersonal violence [20], thereby ensuring a wide spectrum of EC needs. However, current capacity for EC is low in many parts of the Pacific region.

### PECA tool development

The PECA table (see Table 1) was initially developed for the 2009 Timor-Leste national hospital emergency department (ED) Needs Assessment [21] and has since been expanded and refined after repeated use in other Pacific Island contexts. Using an accepted understanding of the components of EC [22, 23] and a patient-centred approach [24], the table begins by mapping a patient journey into categories of EC delivery, and thereby places emphasis on the processes of care. Subsequently, the PECA table seeks to document the ED environment, equipment and human resource details, taking into account less easily observed factors such as staff leadership, confidence and morale. Further quality and process concepts are categorised that cover safety, infection control, patient tracking and flow, data management and communication. Finally, the PECA tool seeks to document the culture, internal and external relationships of the ED.

**Table 1:** Pacific Emergency Care Assessment (PECA) Table outline

Area	Domains	Observations
Demographic data	• Presentations – number, type, distribution • ED relationship with hospital, community	Detailed observations covering all of these areas, including columns / rows for: • Strengths • Weaknesses • Facilitators • Barriers • Identified gaps • Possible solutions • Recommendations
Pre-hospital	• Transport mode, care, referral system	
Triage	• Presentations • Triage nurses – training, supervision • Location and equipment, amenity and safety • Triage system and Clinical resources • Observation of arrived/waiting patients • Timeliness, accuracy, documentation • Provision of 1st aid	
Time to treatment	• Nursing/medical • Notification, sense of urgency, delays	
Initial assessment	• Systematic; teamwork; medical / nursing • Access to lab/radiology; diagnosis and plan	
Review of patient and ongoing care	• Nursing, medical, inpatient (IP) units • Adverse events	
Trauma and resuscitation management	• Trauma response • Teamwork; effectiveness	
Women’s health	• Obstetric care • Sexual violence; privacy	
Paediatric care	• Quality and safety • Environment and equipment • Staff training, communication, IP unit care	
Access to treatment	• Delays; limitations	
Handover	• Within ED; to IP units	
Patient disposition	• To Theatre, IP units, home • Access Block	
Transport of patients	• Staff, timeliness, safety	
Equipment	• Availability; training; maintenance; supply	
Infection control	• Staff and patients • Isolation; cleanliness	
Standard and consistency of care	• Protocols and guidelines • Best practice; EBM; adverse events	
Patient information management	• Documentation; communication • Forms, storage, access, technology use, data for research	
Safety	• Critical incidents; error and review • Staff safety and amenity	
ED staff	• Number and rostering; Human resource use • Education and training level, ED based teaching. Skill mix + supervision • Scope of practice	
Communication	• Between ED staff; ED + hospital staff • Between staff and patients/families	
Culture of ED	• Sense of ownership • Leadership / responsibility • Morale + Staff turnover	
ED design and patient flow	Comprehensive mapping of ED design and how patients move through clinical areas, including • Bottle-neck areas • Access block (all contributing factors) • Patient tracking • Design features; amenity; functionality	Aerial diagrams of current and suggested ED layout with patient flow mapping Recommendations aim to maximise safe and effective use of existing space

Alongside these descriptive domains are sections for comment on strengths, weaknesses, enablers and barriers. When complete, the PECA table aims to provide a comprehensive overview of EC capacity and function embedded in a nuanced understanding of how and why a facility is operating as it is.

### Methodological context

In order for the PECA tool to be both accurate and have local integrity, a non-judgemental and collaborative method of application is essential. We used three complementary methodological approaches in the application of the PECA tool.

#### Strengths-based

Based on the theoretical framework of Appreciative Inquiry [25] used in organisational development, a strengths-based approach enables participants to focus on positive actions, individual skills and group achievements. In Pacific Island EC facilities, where resources are low and attention to facility development has been severely limited over years, it can be easy to become overwhelmed with negative perceptions. By focussing on strengths in individuals and teams, a positive narrative can be created which may then lead to increased levels of local engagement with recommendations for improvement. Such recommendations can be based around the existing strengths of the EC facility.

#### Action research

Action research combines the dual aims of action to bring about change, and research to increase understanding (in the researcher and/or the participants) of why change occurred and the consequences of change [26]. It is participatory, iterative and works to support local community understanding and action, rather than just record information. Although more time consuming than applying a simple checklist, an action research approach engenders local ownership of the project to improve their EC facility and empowers local stakeholders to lead appropriate and sustainable changes. This approach leverages off the high value placed in personal relationship-building in PICs. Relationships of mutual trust must be established before mutual responsibility for positive change can be expected.

#### Ethnography

The practice of ethnography involves immersion in the daily life and activities of the community under study, usually over a long time-period. Typically, this is through regular participation, careful observation, in-depth individual and group interviews and study of ‘artefacts’ related to the community; such as documents, formal records and other public evidence about the community [27]. Ethnography is also a reflexive practice, whereby the researcher becomes aware of their own role and agency in the analysis and interpretation of data collected [28]. In assessing the needs of EC facilities in PICs, we adopted a modified ethnographic approach, necessitated by time limitations, but adhering to the fundamental principles of ethnographic research. This enriched the data collected and enabled deep insights into EC facility function, allowing feedback to local clinicians for their own reflection and learning [29].

### PECA tool implementation

Application of the PECA tool began approximately 2 months prior to in-country data collection in each country case. Context information was sourced from available grey literature, such as national health plans, newspapers, social media sites and reports previously written for government and non-government agencies. Expert informants were interviewed to provide up-to-date health system status and country information, as well as long term historical and cultural context. Relationships with key in-country stakeholders were established.

Research teams in each site comprised an external emergency physician and emergency nurse, with expertise in low resource clinical contexts, EC systems strengthening, EC leadership and education. In one country (Timor-Leste), a local counterpart worked with the research team to facilitate activities and assist with English language translation, which was not required in Kiribati or the Solomon Islands.

The majority of data collection occurred during the brief in-country period, which for cost reasons was confined to 2 weeks. Data was collected and triangulated through direct observation, semi-structured interviews, group interviews, informal conversations and additional in-country access to grey literature.

Primary importance was placed on establishing and enhancing local relationships, and engaging the key local EC leaders through the action research approach. In each site, the national hospital is under constant scrutiny, particularly the ED ‘front door’. There is a risk of monopolising limited time with innumerable stakeholder interviews at the expense of involving those who actually work in the ED and who are a focal audience for the research report. Time is also required to unobtrusively observe, think and feedback to local players. Data collection was iterative, whereby the research team tested observations and ideas with multiple local stakeholders for validity prior to confirmation and entry into the PECA tool. Prior to departure, an open invitation *aide memoire* was delivered in each site for the purpose of summarising preliminary findings to a wide local audience, receiving feedback and gaining local endorsement.

Finally, the analysis of the completed PECA tool, synthesis of findings and recommendations for EC improvement was targeted to two audiences; the government bodies who requested and funded the Needs Assessment, and the local ED clinicians who remain the engaged stakeholders required to lead change. The final report became a resource for local EC leaders and therefore included recommendations that were achievable, realistic and sustainable, as well as satisfying the expectations of national health leaders. A suggested framework for incorporation of the PECA tool data into a final report is shown in Table 2.

**Table 2:** Emergency care needs assessment report framework

Headings	Sub-headings	Content summary
Introduction	Background and Context	
Methods	• Methodological approach • Tools used • Structure and purpose of report	
Findings	Assessment of current function	• Existing strengths • Staff • Systems (processes) • Space (environment & equipment) • Special issues
	Facilitators and barriers	
	Capacity for specific roles/tasks	For example: teaching and clinical supervision
Recommendations	EC development goals	• Staff • Systems (processes) • Space (environment / equip) • Culture, capacity & service
	EC development priorities	Incorporates urgency of issue to be addressed, capacity to improve function and feasibility / achievability
	Strategies for improving EC • In-country / Local • External technical assistance	• No / low cost vs requiring funding • Matched to priorities • Practical and feasible
	Timelines	
Resource considerations		Mindfulness of specific local context and resource issues
Potential models		Suggestions and linkages to complimentary programs, funding streams, networks, other resources
Next steps		
Appendices	List of people consulted	
	Completed PECA table	Completed table serves as a baseline record of capacity and function
	Specific issue recommendations	For example; ED triage, patient flow management, paediatric EC
	Maps of ED (current and potential)	Low cost suggestions for maximising space utility, reducing patient bottle-necks, improving flow

### Follow-up and validation

For each country, the completed PECA table and associated recommendations have been handed over to local ED and government stakeholders. Structured follow-up was not built in to the PECA tool application and methodological framework. Validation was measured through real-time utility of the tool, integrity and relevance of findings, appropriateness of recommendations, future activities and ongoing relationships between researchers and engaged local EC leaders.

The full PECA table, topic guide and semi-structured interview questions are provided in detail as an additional file (see Additional File 1).

## Results

The Solomon Islands Needs Assessment project, performed in 2014, is the main model used to illustrate and discuss outcomes of the PECA tool and methodological framework. Table 3 provides details of this case example, including a brief outline of key findings, recommendations and subsequent EC developments.

**Table 3:** The Solomon Islands needs assessment case example

Findings	Priority Recommendations	Outcomes
Staff • Lack of leadership • Insufficient numbers • Minimal training • Poor morale	Leadership investment Staff recruitment Staff training and development	Two local Emergency Medicine Physicians in leadership roles Australian EM physician and ED Nurse as in-country technical assistance and support 24-h ED medical rostering Local leaders running • regular daily education sessions • annual compulsory competency training to ensure minimum standard of care • rotating overseas professional development opportunities for all staff • new EM Diploma degree • annual ED staff medical checks • junior staff portfolios
Systems • Quality and safety • Poor communication • Limited information systems • Triage o No system o Unsafe practice • Patient Flow o Overcrowding, bottlenecks o Absent management system	Patient flow management systems Patient tracking systems Triage system Quality improvements • Clinical guidelines • Audits • Minimum standards Paediatric care focus	24 h Security staff Recruitment of cleaners Hospital-wide ED Access Block policy and procedures Development of local patient tracking system Development and implementation of the **Solomon Islands Triage Scale (SITS)** ED clinical guidelines Point-of-care testing Weekly audits Team meetings: ED and external Disaster training
Space (environment) • Limited space • Unsafe space • Inadequate equipment	Redesign suggestions • Paediatric area Improve amenity Basic equipment procurement & maintenance	Separate Paediatric ED care space New triage and registration room Air-conditioners, amenity block renovation Individual staff basic equipment packs
Facilitators • Strong nurses • Desire for change • Future potential		Overall outcomes • Good morale • High engagement • ‘Best Department’ award • Future positivity
Barriers • Exhaustion • After-hours issues • Administrative challenges		Risks • Burnout

### PECA tool delivery and feasibility

From a practical use perspective, the PECA performed well. At each site, the PECA tool was able to be almost fully populated with relevant data during the 2 week in-country work and then completed within 4 weeks of return. Because the tool structure was deliberately mapped on to a patient’s journey through the ED, data collection with clinical examples was easily obtained through observation time and conversations with ED clinicians as they performed their daily work. Printed blank copies of the PECA table enabled real-time data capture in environments with unreliable access to electricity and information technology (IT). Daily discussion, feedback from stakeholders and regular team reflection enabled capture of essential details and triangulation of data.

Hard copies of preliminary report summaries were left with key ED and Ministry of Health stakeholders at each site prior to in-country team departure. Full reports were sent widely and without restriction by email at completion of the final analysis and recommendations, which potentially presented a challenge for local stakeholders to read or print given local IT constraints. In the Solomon Islands, local ED leaders printed their own copies of the reports, particularly the Patient Flow Maps and appendices covering potential actions items to address ED Access Block and Paediatric ED Care.

### PECA tool and methodological validity

The validity of the PECA tool and methodological framework can be assessed through its application in the Solomon Islands and the ongoing outcomes generated by this Needs Assessment. Essentially, have the findings from applying the PECA tool made sense for the Solomon Islands ED clinicians in light of what the tool is designed to measure? Furthermore, have the recommendations arising out of the Needs Assessment method provided the Solomon Islands ED clinicians with relevant and practical actions that can improve their ED function? [30].

Table 4 provides a summary of outcome validity across the domains of Face and Content Validity [36]; Construct Validity; Catalytic Validity; and Reliability and Rigour. To elucidate Construct Validity (the ability to identify accurate strengths and gaps and therefore make appropriate recommendations), and Catalytic Validity (“the degree to which the research process re-orients, focuses and energises participants; who transform realities through gaining sufficient knowledge” [37]), a summary of the key findings and recommendations from Timor-Leste (2009) and Kiribati (2011) is provided (Table 5). When compared to the Solomon Islands (Table 3), these examples illustrate how the PECA tool implementation highlighted and addressed local strengths and future concerns.

**Table 4:** Validity outcomes of the PECA tool and methodological framework

**Face and Content Validity**	
• Conforms to structure of other frameworks for defining and assessing facility-based EC (Tanzania [31] and Rwanda [32]) • Adopts a contemporary patient-centred approach • Uses a common language shorthand applied to the essential components of facility based EC [23] • Incorporation of pre-hospital care and emphasis on triage acknowledges the recently developed WHO EC Systems Framework [2] and other WHO EC tools. • Adds new value by including less well measured but equally critical health care characteristics such as communication, leadership and staff morale • Allows insight about more conventional components of ED function, such as effective resuscitation teamwork and therefore leads to more reliable and broader inferences about ED function overall.	
**Construct Validity**	
• Key national hospital, Ministry of Health and Australian Government Aid stakeholders all accepted the core findings of the 2014 Needs Assessment and concurred with the recommendations that prioritised leadership, staff improvements, triage, paediatric care and attention to overcrowding and patient flow • Components from the 2014 Needs Analysis that have been considered and acted upon since delivery to the stakeholders: (Table 3) o development and implementation of a new triage scale [33] o creation of a paediatric EC area within the ED o new protocols for managing ED overcrowding and patient flow o sustained support for local leadership and staff education.	
**Catalytic Validity**	
• Solomon Islands: local ED stakeholders have taken a leadership role in transforming their ED (Table 3). • Each Needs Assessment process has performed as a trigger for locally-led developments • Each Needs Assessment report has provided a tool for subsequent reference and future local energy directed towards ED improvement [34, 35]. (Tables 3 and 5)	
**Reliability and Rigour**	
• Limited ability to measure consistency over time due to single application in each site • Internal consistency and stability of the PECA tool confirmed through inter-observer agreement, triangulation of information, repeated observations at different times and days over the two-week period and iterative feedback from key stakeholders. • Two different nurses performed the Needs Assessment across the three sites thereby confirming inter-rater feasibility and consistency of application • Durability of the PECA tool and methodological approach illustrated through longevity of utility. • Rigour enhanced through reflexivity. Throughout each Needs Assessment project, the researchers / observers reflected on their collegiate and friendship relationships with the participants / observed. • Self-recognition of biases and assumptions aided interpretation of observations.

**Table 5:** Key findings and recommendations from needs analyses in Timor – Leste and Kiribati

Country and Context	Key findings	Priority Recommendations
**Timor-Leste (2009)** **(national referral hospital)** • Post-conflict • Many different service providers • Cuban doctor training program • New ED building • Limited understanding of EC • Strong sense of unity • Change occurs through mentoring and modelling	Lack of triage impacting on quality of care Entrance overcrowding and assessment area bottleneck Poor information management and communication across language/culture Very limited staff training, precarious leadership, sustainability challenges Quality of care limitations Insufficient use of space	Substantial investment in local staff • Identify potential leaders • Provision of ED career structure • Long and short term training • Provide mentors • Short and long term technical assistance Development and implementation of a triage system Clinical guidelines and regular audits Improved formal communication; handover, referral, documentation Basic equipment provision ED re-design suggestions
**Kiribati (2011)** **(national referral hospital)** • Small atoll nation • Very close community • Strong nurse training and nurse culture • Few doctors, some Cuban medical training • Old ED building • Change occurs through senior leadership and consensus collaboration	Inadequate nurse numbers and insufficient EC training Absent medical leadership Very poor environment; not fit for purpose, limited renovation potential, no amenity No triage system Overcrowding and bottlenecks Minimal clinical guidelines or quality standards Minimal equipment and information management resources	Short term • Build ED nursing knowledge and leadership • Minor renovation and re-use of existing space, basic amenity repairs • Development and implementation of a simple triage system • Patient flow and clinical guideline working groups Longer term • Invest in medical ED leadership • Improve IT and data management systems • Source funding for more extensive ED renovation

Recommendations for action differed across each site according to the context, culture, strengths and gaps identified, and were prioritised according to both need and feasibility, for short and long term implementation. To enhance trust and engender long-term local stakeholder commitment, recommendations included simple actions for rapid and successful implementation. In Timor-Leste, with a new ED building but observed insufficient use of space producing overcrowding and bottlenecks, short-term recommendations that triggered early action included simple maps illustrating improved patient flow by re-orientation of clinical care areas. In the absence of medical leadership, but with a strong nursing culture, short-term recommendations in Kiribati included focus on nurse capacity development for clinical care and quality improvement. The Solomon Islands assessment identified an urgent need to improve paediatric EC. Specific and detailed recommendations were provided to local stakeholders outlining immediate and short-term actions involving training, process, equipment and ED environment changes that did not require additional resources nor external drivers.

The PECA model consistently identified leadership as a core issue with different impacts across all sites. For long-term improvements in EC delivery, priority recommendations accepted and acted upon by all local Pacific stakeholders emphasised identification of, investment in, and support for local EC leaders who can inspire and drive change over time.

## Discussion

We describe the first facility-based Needs Assessment tool tailored to the Pacific and applied consistently across three different sites, with the use of case examples in the Solomon Islands to explore validity and reliability of the PECA table and methodological approach. Each Needs Assessment produced context-relevant findings and appropriate, practical recommendations across EC system domains including human resources, environment, equipment, processes of care, culture and leadership. All EC domains interact within a complex system [38]. Therefore, although emphasis for action differed in each site, it is likely that even small improvements in a single domain positively influenced overall EC delivery at each facility. Highlights of subsequent outcomes catalysed by the Needs Assessment process include the development and implementation of the Solomon Islands Triage Scale [33], multidisciplinary EC improvement activities in Kiribati [34] and ongoing program support in Timor-Leste [35].

There are few EC Needs Assessment frameworks in the published literature, mostly from Africa and none from the Pacific region. In Tanzania, using available evidence and a modified Delphi process, researchers developed a set of Structure Standards for Emergency and Critical Care (EaCC) consisting of 104 indicators across the domains of infrastructure, human resources, training, drugs, equipment, routines, guidelines and support services [31]. This tool was then applied across 10 regional and district hospitals and identified gaps in infrastructure, lack of routines and low level of training for EaCC. However, data was often incomplete, findings were generalised and no process measurements were incorporated in to this approach. By contrast, our single facility Needs Assessment approach provides comprehensive data, is specifically tailored to the site and provides detailed and complex information about processes, including facilitators and barriers to effective care delivery.

The Tanzania tool has since been used in Sierra Leone to evaluate the EaCC capacity in seven urban hospitals [39]. At each site, the assessment and tool application lasted from 1 to 2 h and was done by an external researcher in collaboration with a local lead clinician. Although helpful to provide a snapshot of facility capacity and differences between facilities, this approach is prone to bias in self-reporting and allows only limited inferences to be made about daily function and quality of EC. Furthermore, the methodological approach gives little room for local stakeholders to highlight strengths, or gain empowerment to lead positive change.

In 2015, the University of Columbia Systems Improvement at District Hospitals and Regional Training of EC project [32] used their own Emergency Services Resource Assessment Tool to survey all 42 district hospitals in Rwanda. The tool and data collected remain unpublished, but focussed on staffing, infrastructure, medications, equipment, continuing medical education and services available for care of patients with traumatic injuries and emergency conditions.

Researchers in western Kenya used a self-designed data collection instrument and semi-structured, key informant interviews to assess EC capabilities across 60 facilities (ranging from dispensaries, health centres, primary and secondary hospitals) [40]. Assessments were conducted within 1 day, utilising the most senior available facility staff members to answer questions across eight domains: facility demographics, referral services, personnel, economics, supplies and laboratory, trauma, critical care and anaesthesia. Although qualitative information exploring attitudes, morale, staff cooperation and communication was gathered, this model of assessment based on facility leader self-report is open to bias. In contrast, our PECA model collects rigorous qualitative information through interviewing multiple stakeholders, direct observation, iterative discussion and triangulation of several sources of data.

More structured EC data collection tools exist that have been used exclusively or adapted for large-scale, cross-sectional surveys of facility-based capability. The National Emergency Department Inventories survey is a 23-item instrument developed, managed and applied widely across the USA, and in capital cities of China, Nigeria, Colombia and Europe [41–43]. The small scale and need for information beyond general descriptive statistics may make these kinds of tools less relevant for the Pacific region.

Recently, the African Federation for Emergency Medicine (AFEM) Emergency Care Assessment Tool (ECAT) was piloted and refined across four countries; Botswana, Cameroon, Egypt and Uganda [15]. The 71-item tool assesses capacity of EC facilities to perform previously defined signal functions that treat common, life-threatening ‘sentinel’ conditions [44], as well as evaluating barriers to service delivery. The ECAT is completed on-site by a trained administrator interviewing three local participants; one senior doctor, one senior nurse and one other clinical provider, and is designed to assess clinical functional capacity in order to provide a roadmap for facility improvement. Like the PECA tool, the ECAT has a patient care focus, however is designed for broad general facility and system assessment, rather than to collect nuanced details about a single facility function.

The WHO provide checklists for very basic equipment and emergency room capacity under their Integrated Management for Emergency and Essential Surgical Care toolkit [45], which have been used to provide basic information about emergency care capacity in a centre in Sub-Saharan Africa [46]. Current WHO work in Emergency and Trauma Care provides a country-level EC Systems Assessment (ECSA) tool, implemented through a facilitated multi-stakeholder process that aids national policy and planning for EC improvement [47]. Further work to pilot and refine a WHO Emergency Unit Assessment Tool (EUAT) that incorporates the work from Bae et al. [15] on signal function capacity is underway. These tools have relevance for the Pacific region, but provide minimal detail at the individual facility level.

A multi-modal EC assessment approach was piloted in Pakistan in 2008, using three data collection instruments [17]. Separate surveys collected information and perspectives on EC from a range of community members and health care providers. Facility assessment was performed with questionnaire and item inventory during a facility tour, staff in-charge interview and patient log review. This approach, like the PECA, aimed to gather broader insights into the availability and quality of facility-based EC. The quantitative analysis provided useful snapshot information on the status of EC in the Pakistan districts, but was unable to shed detailed light on facility function or priority domains for improvement aside from simple resource availability.

Arguably, the most similar published methodological approach for an EC health system assessment has come from researchers in post-conflict Serbia [48]. In light of the unique and complex EC needs arising out of a damaged and neglected health care system, researchers justified an integrated multimodal assessment as a means to elucidate urgent needs and develop achievable goals specific to the local context. Their particular question was the development of emergency medical services (EMS) in Belgrade rather than the capacity and function of an EC facility. However, through modalities including observational data and detailed qualitative methods, researchers were able to gather and synthesise meaningful information pertaining to the strengths, needs, problems and obstacles of EMS, and therefore identify priorities for action. Although not the same as the ethnographic and action-research approach of the PECA methodology, this research has similar strengths of identifying context-specific issues and engaging local stakeholders in the development process. Similarities are also apparent in the post-conflict milieu, with both Timor-Leste and the Solomon Islands emerging from destructive and violent civil unrest.

### Lessons learnt

#### Strengths

The comprehensive, free-text structure combined with patient-centred framework allows the PECA tool to collect detailed and nuanced information about both capacity and real-life function of an EC facility. Whilst conventional EC assessment tools include checklists with an emphasis on whether facilities have the necessary equipment for effective life-saving care, they cannot always comment on whether the equipment is used at the right time, in the right way, for the right patient. The PECA table combined with its action-based and ethnographic application attempts to fill this gap by including observations and critique from which global level of EC function may be inferred.

The qualitative methodological focus on relationships and local stakeholder agency gives the PECA findings more than just an academic status report, but implies ongoing engagement, partnership and action for positive change.

### Limitations

Key limiting factors to widespread use of the PECA tool and approach include time and lack of generalisability. It is for a detailed analysis of a single site, not a comprehensive overview of the status of facility-based EC in a region. In the Pacific context, where there is often only one referral hospital for the entire country or region, this approach is well suited. Time taken in preparation, in-country work, synthesis and follow-up is not feasible for larger scale EC Needs Assessments.

Furthermore, this approach brings expectations and responsibility to act. It is unethical to engage local stakeholders in an action research project without a commitment or the ability to follow-through. This can generate tension between funders, researchers and local EC clinicians if priorities change. Researchers using the PECA approach have a responsibility to provide a resource that empowers local leaders to prioritise, plan and enact their own EC development within their local resource constraints.

Work to create and maintain positive relationships between researchers and local EC clinicians is essential to the success of the PECA approach. Whereas impartial outsiders may perform alternative models of EC Needs Assessments, this model necessitates a relational approach. Quality of data depends on engagement with local stakeholders and by nature is subjective. The tension between creating open, safe and affirmative communication with local informants through a friendly, supportive relationship and minimising bias in data collection must be reconciled through careful and collaborative reflexive practice.

Finally, there is no evaluation component built in to any of the PIC Needs Assessments projects. From a comparative perspective, follow-up studies in each site should also adopt a PECA methodology, but are time and resource-heavy. More simple EC Needs Assessment tools allow for rapid follow-up data collection and allow for measures of development over time. In this paper, we rely on stakeholder report of subsequent EC developments to illustrate impact of the PECA approach.

## Conclusion

In PICs, where human and other resources are limited and health systems rely on safe and effective EC, a strengths-based model of analysing needs for EC improvement is recommended. A PECA-style method that prioritises relationships, participant observation and community appraisal can trigger lasting transformational change through high level of local stakeholder engagement. Whilst developed for the PIC context, testing this approach in other global low resource health environments would add validity and credibility. Future work that combines the strengths of the PECA approach (qualitative, relational, action-orientated, positive) with an adapted, standardised EC assessment instrument is the next step. Substituting lengthy and costly field time with remote communication amongst key stakeholders may address the challenge of time. Improving quality of data collected within a reduced time frame can lead to wider applicability of an EC Needs Assessment tool. Incorporating evaluation and meaningful outcome measures into such an assessment tool would provide data that may then guide policy and practice for EC development globally.

## Supplementary information

**Additional file 1.**

## Data Availability

Data sharing is not applicable to this article as no datasets were generated or analysed during the course of the study. In order to respect the original purpose of each Needs Assessment, the detailed reports for each country are not provided and will not be shared in any publically available repository. These reports are for the local stakeholders and accessing the detailed information contained within them is not necessary for the interpretation of our manuscript.

## Abbreviations

EC: Emergency care
PICs: Pacific Island Countries
PECA: Pacific Emergency Care Assessment
WHO: World Health Organisation
ED: Emergency department
IT: Information technology
EaCC: Emergency and critical care
AFEM: African Federation for Emergency Medicine
ECAT: Emergency Care Assessment Tool
ECSA: Emergency Care Systems Assessment
EUAT: Emergency Unit Assessment Tool
EMS: Emergency Medical Services
