# Supplementary material for: A Pacific needs analysis model: a proposed methodology for assessing the needs of facility-based emergency care in the Pacific region
Source: BMC Health Serv Res. 2020 Jun 19;20:560. doi: 10.1186/s12913-020-05398-w (PMC7304213; doi:10.1186/s12913-020-05398-w)
Supplement: Supplementary file 1 — Additional file 1. [file 12913_2020_5398_MOESM1_ESM.pdf]

Pacific Emergency Care Assessment (PECA) Table

Emergency Department

XX Hospital (City, Country)

Area	Observations
**Background**<br><br>Number of presentations<br><br>Types of presentations<br><br><u>Number of staff</u><br>Medical<br><br>Nursing<br><br>Support<br><br><u>Number of beds</u><br>ED<br><br>Hospital<br><br>*Facilitators / strengths*<br>*Barriers / weaknesses*<br>*Recommendations*	

**Pre Hospital Care**<br><br>Pre-hospital system<br><br>Ambulances / other transport<br><br>**Referrals GPs and other hospitals**<br><br>*Facilitators / strengths*<br>*Barriers / weaknesses*<br>*Recommendations*	
**TRIAGE**<br>**Presentations**<br>Numbers<br><br>Types<br><br>*Facilitators / strengths*<br>*Barriers / weaknesses*<br>*Recommendations*<br><br>**Triage nurses**<br>Training<br><br>Experience<br><br>Seniority	

Facilitators / strengths<br>*Barriers / weaknesses*<br>*Recommendations*<br><br>**Location of triage**<br><br>*Facilitators / strengths*<br>*Barriers / weaknesses*<br>*Recommendations*<br><br>**Physical area / Design**<br><br>• Space<br>• For staff<br>• For patients / family<br>• Safety<br>• Equipment<br>• Clinical Resources available at triage<br><br>*Facilitators / strengths*<br>*Barriers / weaknesses*<br>*Recommendations*<br><br>**Triage system**<br><br>Use of current triage guidelines	

Facilitators / strengths<br>*Barriers / weaknesses*<br>*Recommendations*<br><br>**Observation of arriving patients**<br><br>*Facilitators / strengths*<br>*Barriers / weaknesses*<br>*Recommendations*<br><br>**Timeliness to triage**<br><br>*Facilitators / strengths*<br>*Barriers / weaknesses*<br>*Recommendations*<br><br>**Allocation of triage category**<br><br>•Appropriate allocation<br>•Timeliness of allocation<br>•Consistency<br><br>*Facilitators / strengths*<br>*Barriers / weaknesses*<br>*Recommendations*	

**Identification of primary problem**<br><br>*Facilitators / strengths*<br>*Barriers / weaknesses*<br>*Recommendations*<br><br>**Provision of first aid**<br><br>*Facilitators / strengths*<br>*Barriers / weaknesses*<br>*Recommendations*<br><br>**Information recorded at triage**<br><br>*Facilitators / strengths*<br>*Barriers / weaknesses*<br>*Recommendations*<br><br>**Facilitation of movement of patients into appropriate area in the department and communication with staff.**<br><br>*Facilitators / strengths*<br>*Barriers / weaknesses*<br>*Recommendations*	

**Movement of patients into the department**<br><br>Promptness<br><br>Cubicles<br><br>Waiting areas<br><br>*Facilitators / strengths*<br>*Barriers / weaknesses*<br>*Recommendations*	
**Time to treatment**<br><br>Nursing care<br><br>Medical care<br><br>Notification of doctors<br><br>Sense of urgency<br><br>Delays<br><br>*Facilitators / strengths*<br>*Barriers / weaknesses*<br>*Recommendations*	

**Initial assessment**<br><br>Systematic assessment<br><br>Medical<br><br>Nursing<br><br>Teamwork<br><br>Diagnosis / Major problem identified<br><br>Access to pathology, radiology and results<br><br>Documentation<br><br>*Facilitators / strengths*<br>*Barriers / weaknesses*<br>*Recommendations*	
**Review of patient and ongoing care**<br><br>ED nursing staff<br><br>ED medical staff	

Inpatient Units<br><br>*Facilitators / strengths*<br>*Barriers / weaknesses*<br>*Recommendations*	
**Trauma and resuscitation management**<br><br>Trauma response<br><br>Resuscitation response<br><br>Team work<br><br>*Facilitators / strengths*<br>*Barriers / weaknesses*<br>*Recommendations*	
**Care of paediatric patients**<br><br>Assessment<br><br>Management<br><br>Space / equipment for care<br><br>Role of IP unit	

Facilitators / strengths<br>*Barriers / weaknesses*<br>*Recommendations*	
**Women's health**<br><br>Maternity<br><br>Sexual / Family violence<br><br>*Facilitators / strengths*<br>*Barriers / weaknesses*<br>*Recommendations*	
**Access to all EC treatment**<br><br>Delays<br><br>Unavailable<br><br>*Facilitators / strengths*<br>*Barriers / weaknesses*<br>*Recommendations*	
**Handover**<br><br>**Medical**<br>• Shift to shift<br>• To Units	

**Nursing**<br>• Shift to shift<br>• To ward<br><br>*Facilitators / strengths*<br>*Barriers / weaknesses*<br>*Recommendations*	
**Disposition**<br><br>Access to theatre<br><br>Access to inpatient beds<br><br>*Facilitators / strengths*<br>*Barriers / weaknesses*<br>*Recommendations*	
**Transporting of patients**<br><br>To ward<br><br>To X-ray department<br><br>*Facilitators / strengths*<br>*Barriers / weaknesses*<br>*Recommendations*	

**Activity levels**<br><br>High<br><br>Low<br><br>After hours and overnight<br><br>Relationship to staffing<br><br>*Facilitators / strengths*<br>*Barriers / weaknesses*<br>*Recommendations*	
**Department design**<br><br>Flow of patients<br><br>Control of entry<br><br>Space / structure<br><br>Amenity<br><br>Resuscitation areas<br><br>High acuity areas<br><br>Low acuity / fast-track	

Procedure areas<br><br>Observations areas<br><br>Staff / teaching areas<br><br>(accompanied by maps of ED and patient flow diagrams through the department)<br><br>*Facilitators / strengths*<br>*Barriers / weaknesses*<br>*Recommendations*	
**Patient tracking**<br><br>Into the department<br><br>Through the department<br><br>Out of the department<br><br>*Facilitators / strengths*<br>*Barriers / weaknesses*<br>*Recommendations*	
**Equipment**<br><br>Availability	

Appropriateness<br><br>Training<br><br>Maintenance<br><br>*Facilitators / strengths*<br>*Barriers / weaknesses*<br>*Recommendations*	
**Infection control**<br><br>Staff and patients<br><br>Isolation<br><br>Cleanliness<br><br>*Facilitators / strengths*<br>*Barriers / weaknesses*<br>*Recommendations*	
**Standard and consistency of care**<br><br>Existence and use of protocols<br><br>Evidence of best practice	

Factors that impact on level and quality of care<br><br>*Facilitators / strengths*<br>*Barriers / weaknesses*<br>*Recommendations*	
**Data / document management**<br><br>Data collection and storage<br><br>Use of IT<br><br>Patient notes<br><br>Facilitators / strengths<br>Barriers / weaknesses<br>Recommendations	
**Safety**<br><br>Critical incidents<br><br>Error and review process<br><br>Sharps / blood<br><br>Staff Safety	

Facilitators / strengths<br>*Barriers / weaknesses*<br>*Recommendations*	
**Level of staff education and training: Nursing**<br><br>Undergraduate training<br><br>Specialty training<br><br>In-house training<br><br>*Facilitators / strengths*<br>*Barriers / weaknesses*<br>*Recommendations*	
**Medical staff education and training**<br><br>Medical students<br><br>Specialty training<br><br>In-house education<br><br>*Facilitators / strengths*<br>*Barriers / weaknesses*	

Recommendations	
**Nursing Staffing levels**<br><br>Number of staff per shift<br><br>Shift times<br><br>Handover<br><br>Patient ratios<br><br>Skill mix<br><br>*Facilitators / strengths*<br>*Barriers / weaknesses*<br>*Recommendations*	
**Medical Staffing levels**<br><br>Number of staff per shift<br><br>Shift times<br><br>Skill mix<br><br>Supervision of junior staff<br><br>*Facilitators / strengths*	

Barriers / weaknesses<br>*Recommendations*	
**Nursing roles**<br><br>Scope of practice<br><br>Nursing structure<br><br>Autonomy<br><br>*Facilitators / strengths*<br>*Barriers / weaknesses*<br>*Recommendations*	
**Utilisation of Human resources**<br><br>Medical staff<br><br>Nursing staff<br><br>Support staff<br><br>Medical students<br><br>*Facilitators / strengths*<br>*Barriers / weaknesses*<br>*Recommendations*	

**Communication**<br><br>Between ED nursing staff<br><br>Between ED medical staff<br><br>Staff and patients<br><br>Between medical and nursing staff in the ED<br><br>Between ED staff and hospital administration<br><br>Between ED and radiology and pathology<br><br>Between ED and IP units<br><br>*Facilitators / strengths*<br>*Barriers / weaknesses*<br>*Recommendations*	
**"Culture" of ED**<br><br>Sense of ownership and unity<br><br>Responsibility / leadership	

Staff turnover<br><br>Sick leave / punctuality<br><br>Morale<br><br>*Facilitators / strengths*<br>*Barriers / weaknesses*<br>*Recommendations*	
**Other issues / observations**<br><br>**Features / function unique to this ED**<br><br>**Features / function unique to this hospital**	

Pacific Emergency Care Needs Assessment
XX Hospital (City / Country)

Topic Guide and Semi-Structured Interview Questions

QUESTIONS USED IN PRE-DEPARTURE INTERVIEWS (external visitors / stakeholders)

What is your perception of the function of the ED at XX?
- What are the strengths?
- What are the weaknesses?
- What about the internal systems such as triage and patient flow?

How does the ED fit within the XX hospital / health system?
How does the ED at XX fit within (City) and (Country) nationally, and how is it perceived?
What are the internal and external factors affecting function, fit and perception?
What is your observation of the staff of the ED (medical and nursing)?
- Skills and knowledge
- Team spirit and commitment

What do you think the priorities for development of the ED are?
What strategies for development would be suitable?
Would a long term ED medical / nurse expert support / advisor be suitable and acceptable? What factors would need to be in place for such a placement to work?

QUESTIONS FOR ED STAFF (AND OTHER XX Hospital STAFF)

Assessing function

What is the role/purpose of the ED at XX Hospital?
What is your role within the ED?
- How long have you worked here in this role or other ED roles?

How do you think the ED fits in with the rest of the hospital?
What do other hospital staff think of the ED?
What do the people of (City / Country) think of the ED at XX Hospital?
How well do think the ED at XX Hospital functions?
Is it a good place to work?
- Why / Why not?

Are you able to care for patients in the way you would like to?
What stops you from being able to give the care you would like to give?
What are the good things about this department?

Why are they good and why do they work?
What are you most proud of in this ED?
What things don't work about this department?
Why don't they work?
What would you like to be able to do but cant?
Why can't you?
What are you least proud of in this ED?
Can you think of any examples of something that went well in the ED?
Why did it go well? What worked?
Can you think of examples of something that didn't go well in the ED?
Why didn't it go well? What went wrong?
Is the ED well managed?
Why / why not?
Do others in the ED agree with you? Does everyone think the same way?

Strategies for development / change

Does the ED at XX Hospital need to change?
Is this a priority?
What are the things about the ED that need to change?
What are the priorities?
If you could change one thing in the ED, what would it be?
Have things changed before in this ED?
Were things done differently before?
Have there been any improvements in the ED?
Have things got worse in the ED?
How did the change happen? What was the difference?
How do you think things could change in the ED now?
Is it to do with staffing?
Is it to do with training?
Is it to do with management / leadership?
Is it to do with the environment / equipment?
Is it to do with ED / hospital / staff habits or culture?
How do you think the ED could be developed?
What role could you play?
What help is required?
How long would it take?
What is your vision for the ED in the future?
How would you like to see it function?
What would you like to be able to do in your job to help look after sick patients?

What would you like patients and their families to be able to experience when they come to the ED?
How could things change so that these things could happen?
What steps need to happen so that things could change?
If you were asked to develop the ED, how would you do it?
    What would you concentrate on?
    What is the most important step or factor in beginning the development?

Needs for technical assistance (optional Qs)

How do you think the ED could be developed?
What role could you play?
What help is required?
How long would it take?
Are there skills / knowledge that you don't have now that you think you need to do your job better?
Are there certain people (doctors/nurses/administrators etc) that you think would make a difference to the functioning of the ED?
Is there certain equipment or environmental things that would make a difference to the functioning of the ED?
If an emergency specialist doctor and nurse (from overseas) came to work here, would it make a difference?
    How long would they need to work for?
    What tasks should they do?
    How could they help?
    What skills should they have?
    Cultural / Language preferences?

QUESTIONS FOR XX Hospital LEADERS (Admin and clinical)

What is the role/purpose of the ED at XX Hospital?
How do you think the ED fits in with the rest of the hospital?
What do staff in your area think of the ED?
What do the people of (City / Country) think of the ED at XX Hospital?
What is the role / purpose of EDs at provincial or district referral hospitals and clinics?
How well do think the ED at XX Hospital functions?
What do you see as the factors impacting on the functioning of the ED?
How are staff distributed around the XX Hospital and what proportion of them are for the ED?
Is the ED a site of training and supervision for junior clinical staff?

What is in place for new (Pacific Is Country) doctors returning from medical training in (other country)?
Do you think developing the ED is a priority at XX Hospital or other provincial or district referral hospitals?
What is your vision for the ED in the future?
What are the priorities for improvement?
How would you develop the ED at XX Hospital?
How have other departments at the hospital developed?
What are the key components of development / change at the hospital?
    Is it to do with staff?
    Is it to do with training and education?
    Is it to do with leadership or management?
    Is it to do with environment and equipment?
What has worked well so far in building up and developing the hospital?
What hasn't worked? Why / Why not?
What external help is required to develop the ED at XX Hospital?
How long for?
What would external help look like?
    Staff?
    Training programs?
    Equipment?
What qualities and skills are required?
What past models of success are there of external help at XX Hospital?
What support would the external help (if a clinical specialist) receive?
    Is there capacity for a counterpart to be trained?
What would happen once the external help left?
Does the hospital have a long term commitment to developing the ED and establishing the practice of emergency medicine in (Pacific Is Country)?
Are you aware of any models for developing EM and emergency care in other countries, or in this region?
Do you have a particular country or regional preference / alignment with regards to longer term specialist training and development?
What networks already exist and are currently being established?

QUESTIONS FOR Ministry of Health and OTHER STAKEHOLDERS

What is the role/purpose of the ED at XX Hospital?
How do you think the ED fits in with the rest of the hospital?
What do the people of (City / Country) think of the ED at XX Hospital?

What is the role / purpose of EDs at provincial or district referral hospitals?
How does emergency care fit with the national health priorities of the (Pacific Island Country)?
How well do think the ED at XX Hospital functions?
What do you see as the factors impacting on the functioning of the ED?
Do you think developing the ED is a priority at XX Hospital or other provincial and district referral hospitals?

Why / Why not?

Does emergency care form part of your disaster planning for (Pacific Island Country)?
Do you have a vision for the ED in the future?
What role could you / your organisation play in developing the ED or emergency care services and systems in (Pacific Island Country)?
What do you perceive as the key components to developing emergency care systems in (Pacific Island Country)?
How, in your experience has effective change / development been implemented so far in (Pacific Island Country)?

Is it staff / personnel?
Is it training and education?
Is it leadership and / or management?
Is it environmental or equipment driven?

Would you have a long term commitment to developing ED services and emergency care systems at XX Hospital and in (Pacific Island Country)?
Are there other models for developing EM, EDs and emergency care that you are aware of or have had experience of?

What are the strengths / weaknesses of other models?
